# Hydration Status and Cardiovascular Function

**DOI:** 10.3390/nu11081866

**Published:** 2019-08-11

**Authors:** Joseph C. Watso, William B. Farquhar

**Affiliations:** Department of Kinesiology and Applied Physiology, University of Delaware, Newark, DE 19713, USA

**Keywords:** hypohydration, vascular function, sympathetic nervous system, blood pressure regulation

## Abstract

Hypohydration, defined as a state of low body water, increases thirst sensations, arginine vasopressin release, and elicits renin–angiotensin–aldosterone system activation to replenish intra- and extra-cellular fluid stores. Hypohydration impairs mental and physical performance, but new evidence suggests hypohydration may also have deleterious effects on cardiovascular health. This is alarming because cardiovascular disease is the leading cause of death in the United States. Observational studies have linked habitual low water intake with increased future risk for adverse cardiovascular events. While it is currently unclear how chronic reductions in water intake may predispose individuals to greater future risk for adverse cardiovascular events, there is evidence that acute hypohydration impairs vascular function and blood pressure (BP) regulation. Specifically, acute hypohydration may reduce endothelial function, increase sympathetic nervous system activity, and worsen orthostatic tolerance. Therefore, the purpose of this review is to present the currently available evidence linking acute hypohydration with altered vascular function and BP regulation.

## 1. The Physiology of Hypohydration

Hypohydration is defined as a body water deficit caused by acute or chronic dehydration [1]. While extensive research has been conducted to identify the “elusive daily water requirement”, well summarized by Armstrong and Johnson [2] within this special issue, acute hypohydration studies have provided important insight into the integrative physiology of water balance in humans. Human hypohydration can be elicited experimentally through the use of water restriction, prolonged exercise, heat stress, diuretic administration, or a combination of methods [3,4,5,6,7,8,9,10,11,12,13,14,15,16,17,18,19,20,21,22,23,24,25,26,27,28,29,30,31]. In response to hypohydration-induced reductions in plasma volume and increases in plasma sodium ([Na^+^])/osmolality, the renin–angiotensin–aldosterone system becomes activated, thirst sensations increase, and arginine vasopressin (AVP, also referred to as anti-diuretic hormone) release increases [20,32,33,34,35,36,37,38,39,40]. A low extracellular fluid volume is sensed in the walls of the afferent arterioles proximal to the glomeruli and causes juxtaglomerular cells to secrete renin, which initiates a cascade culminating in increased circulating angiotensin II (Ang II) and aldosterone concentrations acting to increase [Na^+^] and water retention. Central [Na^+^] sensing, which may be distinct from osmo-sensing [41], occurs in circumventricular organs including the organum vasculosum of the lamina terminalis (OVLT) and subfornical organ (SFO) because both brain areas lack a complete blood–brain barrier (BBB) [42]. Specialized mechanical-stretch transient receptor potential vanilloid (TRPV) cation channels are one potential candidate thought to participate in osmo-sensing [43]. Nevertheless, these signals are communicated through neuronal projections to the median preoptic nucleus (MnPO) before activating thirst-promoting neurons in the paraventricular nucleus (PVN) of the hypothalamus via acid-sensing ion channel 1a (ASIC1a) by H^+^ ions exported from Na_x_-positive glial cells [44]. These signals are then 1) relayed to the lateral hypothalamus as well as the paraventricular hypothalamus and thalamus [45], and 2) stimulate AVP release from the posterior pituitary gland from upstream communication with the PVN and supraoptic nuclei [34,46]. Increased thirst sensations promote water intake [45,46]. Increased plasma [AVP] stimulate aquaporin-2-mediated water reabsorption from the luminal surface of renal collecting ducts to promote water retention [47]. Together, these integrated responses aim to restore body water homeostasis.

The following sections will discuss recent findings related to hypohydration and cardiovascular function. When applicable, we will mention the methods used to induce hypohydration (e.g., heat, exercise, fluid restriction, or diuretic) in humans because these methods have different side effects (e.g., diuretics promote iso-osmotic hypovolemia whereas heat stress promotes hyper-osmotic hypovolemia) [48]. Finally, for human hypohydration studies, we will report the resultant body mass deficit as the severity of hypohydration is defined as follows: mild hypohydration (1 to 5% body mass deficit), moderate hypohydration (5 to 10% body mass deficit), and severe hypohydration (>10% body mass deficit) [1].

## 2. Clinical Relevance

As early as 1933, insufficient body water stores were identified as a primary factor for heat exhaustion and fatigue, with scientists concluding, “Most people need the advice: Drink more water” [49]. Approximately a decade later, two scientists deprived themselves of water for over three days and became, “temperamental, hollow, and pale.” Despite noting, “dry mouths, husky voices, and difficulty swallowing,” the authors were, “never unbearably thirsty.” While this prolonged fluid deprivation would now have major ethical concerns, this experiment serves as an early example of how a lack of fluid intake alone can elicit substantial (~5%) reductions in body mass and large (~10%) increases in plasma [Na^+^] [9]. 

While one 2019 report acknowledges that the field has yet to agree on the biomarker(s) and cutoff(s) that define euhydration (optimal total body water content [1]), only 13 to 51% of individuals studied (depending on sex, age group, and disease status) met the authors hydration criteria [50]. Additionally, Americans are not meeting water intake recommendations [51], which is alarming as inadequate water intake is associated with obesity [52] and predicts greater future risk for developing cardiovascular disease [53], the leading cause of death in the United States [54]. Additionally, suboptimal water intake has been demonstrated to enhance serum- and glucocorticoid-inducible kinase 1 activity (SGK1), which participates in the pathophysiology of a number of disease states including hypertension, thrombosis, stroke, and cardiac fibrosis [55]. Further, there are data demonstrating a positive association between plasma [Na^+^] and 10-year risk of coronary heart disease in participants from the Atherosclerosis Risk in Community (ARIC) Study [56]. Some [57,58,59] but not all [60] epidemiological evidence suggests an association between greater plasma [Na^+^] and increased arterial blood pressure (BP). 

While increasing age has been demonstrated to be associated with indices of reduced hydration status [61], the findings from one 2019 study suggest that increasing age is *not* associated with indices of reduced hydration status [50]. Nevertheless, there are several physiological reasons that old adults are less likely to have optimal hydration status including lower basal total body water [62], altered extracellular fluid sensing [63], blunted hormonal (e.g., AVP) responses [64,65], and impaired kidney function [66]. However, even within young healthy individuals, several investigations have provided evidence that acute hypohydration can significantly affect physiological function.

**Figure 1:**
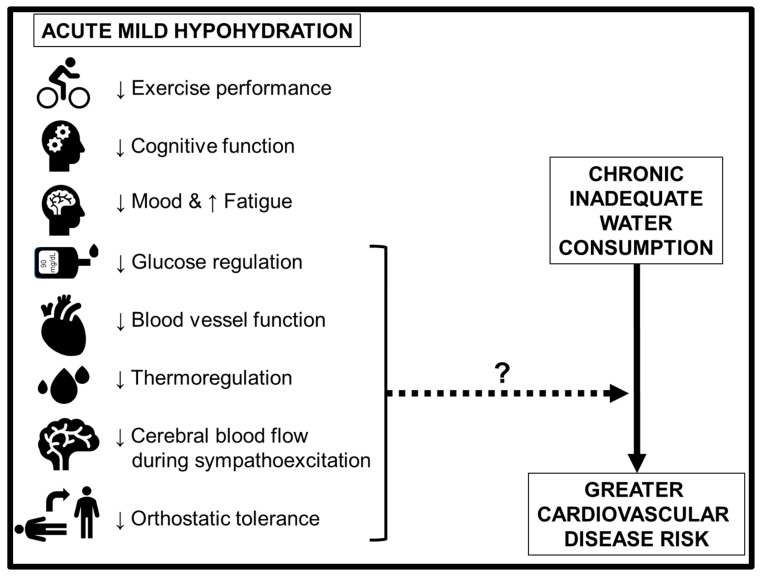
Summary of the physiological consequences of acute mild hypohydration in healthy humans. Further research is necessary to determine whether and how these acute effects influence the poor cardiovascular health outcomes associated with chronic inadequate water consumption. ↓, impaired or reduced; ↑, increased.

For example, there are well-appreciated deleterious effects of acute hypohydration including reduced exercise performance [3,4,10,12,13,14,16,67,68,69,70,71,72,73,74,75,76,77,78,79,80,81,82,83,84,85,86,87,88], worsened mood [18,89,90,91], impaired cognitive function [19,20,21,91,92], altered thermoregulatory function [73,74,80,82,84,87,93,94,95,96,97,98,99,100,101,102,103,104,105,106,107,108], and decreased glycemic regulation [11,109] (Figure 1). Chronic systemic hypohydration is a proposed pathogenic factor for hypertension, venous thromboembolism, fatal coronary heart disease, stroke [110]. However, there are relatively few randomized trials examining the effects of acute or chronic mild hypohydration on vascular function and BP regulation. Importantly, reduced vascular function [111,112,113,114], high resting BP (i.e., hypertension) [115], high BP variability [116,117,118,119,120], orthostatic intolerance [121,122], and exaggerated BP responses during exercise [123,124,125,126,127] are independent clinical predictors for adverse cardiovascular health outcomes. Thus, given the clinical relevance of this area of research, the purpose of this review is to present the currently available evidence on the effects of acute mild hypohydration on vascular function and BP regulation.

## 3. Vascular Health and Function

### 3.1. Inflammation

As discussed above, hypohydration is characterized by elevated plasma [Na^+^]. Dmitrieva et al. [56] demonstrated that Human Primary Umbilical Endothelial Cells (HUVEC) exposed to media with increasing [NaCl] (several concentrations ranging from 270 to 380 mOsm/kg H_2_O) for 4 days were found to have significant increases in the mRNA expression of several pro-inflammatory mediators including vascular cell adhesion molecule 1 (VCAM-1), endothelial-leukocyte adhesion molecule 1 (E-selectin), and monocyte chemoattractant protein 1 (MCP-1). The authors performed additional experiments in rodents to elucidate the effects of physiological increases in [Na^+^] in vivo. Nine days of water restriction increased serum [Na^+^] by ~5 mM without altering body mass and the increased mRNA expression of VCAM-1, E-selectin, and chemokine MCP-1 in several tissues (e.g., liver, spleen, kidney). Additionally, VCAM-1 protein expression was increased in endothelial cells of liver capillaries and coronary arteries. Because long-term inflammation could increase the risk for the development of atherosclerotic lesions, the authors performed a final experiment in mice. ApoE^-/-^ mice were fed a Western diet for 7–9 weeks with water intake ad libitum or restricted. The authors demonstrated a greater development of atherosclerotic lesions in the aortic root and thicker walls of their coronary arteries in water-restricted mice, suggesting prolonged water restriction may contribute to unfavorable vascular health [56]. Costa et al. [128] sought to determine whether hypohydration worsened the inflammatory profile in healthy humans. In randomized crossover fashion, participants either maintained euhydration or had water restricted (hypohydration, ~3% reduction in body mass) while running at an ambient temperature (25 °C) on two separate occasions. The authors reported modest disturbances in gastrointestinal integrity and function as well as in-vitro neutrophil functional responses, but no effect on post-exercise total or differential leukocyte counts, endotoxemia, or cytokinemia following the hypohydration trial. The authors suggested that when taken together, this mild degree of hypohydration was insufficient to induce immune functional or cytokine responses of clinical significance [128]. While this human study was carried out with healthy endurance-trained adults, future studies investigating the influence of reduced water intake alone (i.e., not exercise induced) on the immune system in preclinical and clinical populations are warranted.

### 3.2. Endothelial Function

Endothelial dysfunction is a clinically significant marker of cardiovascular health [111,112,114]. There are cellular studies demonstrating that hypernatremia (high Na^+^ concentrations in fluid) results in degradation of the endothelial glycocalyx, which may also contribute to impaired endothelial responsiveness to shear stress [129]. Arnaoutis et al. [27] sought to determine whether hypohydration impairs peripheral artery vasodilatory function in healthy young male adults. A ~2% reduction in body mass was achieved with 100 minutes of low-intensity (70% of maximal heart rate) walking in mild heat (31 °C) with a 500-mL water intake limit for the remainder of the day. Compared to the same perturbation without a water intake limit, participants demonstrated reduced flow-mediation dilation (FMD, an assessment of endothelial-dependent vasodilatory function) in the water-restricted state [27]. The authors acknowledge the limitation that blood viscosity was not assessed but could have been increased during hypohydration. This is relevant because some published data suggest blood viscosity does affect FMD values [130] but other data suggest that shear rate (blood velocity/vessel diameter) is a weaker correlate of FMD than shear stress (blood viscosity*blood velocity/vessel diameter) [131]. Nevertheless, shear stress was not different between conditions at baseline or during hyperemia [27]. Additionally, while it is unlikely that FMD values in the present study [27] were affected by exercise 24 hours prior [132], future investigations examining the effects of water restriction alone on endothelial function are warranted. Finally, these future studies should be carried out in both male and female adults.

### 3.3. Arterial Stiffness

Aortic stiffness expressed as aortic pulse wave velocity (PWV) is a strong predictor of future cardiovascular events and all-cause mortality [133]. One study examined whether hypohydration-induced via 24-hour fluid restriction or acute heat stress (49 °C water in perfused suit) affects PWV in healthy humans [26]. Caldwell et al. reported that 24-hour fluid restriction in young female adults elicited a ~1% reduction in body mass and reduced central, but not peripheral, PWV compared to the euhydrated control condition. In the same article, a cohort of young male adults underwent whole-body heating to increase rectal temperature +1.0 °C and had fluid intake restricted, resulting in a ~2% body mass loss relative to when participants repeated the whole-body heating on a separate occasion but ingested water to prevent body mass loss. Despite the presence of mild hypohydration in the water-restricted state, participants had similar reductions in peripheral PWV throughout heat stress regardless of condition. Finally, central PWV did not change during acute heat stress in either group [26]. These findings suggest that fluid restriction-induced hypohydration reduces central PWV and heat stress-induced hypohydration does not change central PWV. The authors purposefully designed the study to include homogenous groups because their pilot testing demonstrated large sex-related differences in resting PWV values that would have made the interpretation of findings much more difficult as biological sex could be as important of a factor for altering PWV as the technique used to elicit hypohydration [26]. Thus, it remains unclear whether fluid restriction in males or heat-stress and water restriction in females elicits similar responses.

### 3.4. Cutaneous Vascular Function

There is evidence that mild hypohydration (at either ~1% [22] or ~3% [23] body mass loss) impairs cutaneous vasodilation during exercise in the heat following fluid restriction in healthy male adults. More recent work suggests that hypohydration-induced reductions in skin blood flow are at least partially attributed to altered postsynaptic function in healthy young male adults. This hypothesis is supported by evidence that more methacholine chloride (an endothelium-dependent vasodilator) is required to achieve the drug concentration that provides half of the maximal response (EC_50_) during hypohydration to ~2% body mass loss via exercise in the heat following fluid restriction compared to euhydration [25]. While only one [22] of these particular three studies examining vascular function [22,23,25] reported greater increases in body temperature in the hypohydrated state, several other studies have found hydration status to affect thermoregulatory function [73,74,80,82,84,87,93,94,95,96,97,98,99,100,101,102,103,104,105,106,107,108,134]. As a result, specific guidelines for hydration status have been set in certain populations, such as industrial workers in the heat [135], to minimize the potential for heat-stress and hypohydration-induced increases in cardiovascular strain and potential risk for adverse cardiovascular events. For further discussion on this topic, the reader is directed to several excellent reviews on the interactions between hydration and thermoregulation [70,71,72,103,136,137,138]. Additional work in this area is warranted, particularly studies that include female adults.

### 3.5. Circulating Factors 

During hypohydration, elevated plasma [Ang II] elicits vasoconstriction in small arterioles to increase total peripheral resistance [139] and is thought to contribute to endothelial dysfunction [140]. Specifically, Ang II infusion elicits endothelial dysfunction in rodents [141,142,143,144] and stimulates NADPH oxidase (NOX)-mediated increases in reactive oxygen species (ROS) in smooth muscle cells from human resistance arterioles [145,146]. Further, data from rodent models suggest that hypohydration increases Ang II receptor density and affects neuronal nitric oxide synthase (nNOS) mRNA expression [147]. These data suggested that Ang II blockade may reduce oxidative stress and improve vascular function in humans. In support of this hypothesis, one study reported that Ang II blockade (candesartan) reduced oxidative stress and improved FMD in hypertensive adults [148]. More recent evidence suggests that Ang II blockade reduces inflammation and improves peripheral vascular function in healthy and clinical populations [149,150,151,152,153]. For an extended discussion on the effects of Ang II blockade on vascular function in hypertensive adults, the reader is directed to a recent review article on this topic [154].

Results from one rodent study suggest that increased plasma [AVP] during hypohydration contributes to the production of ROS, elicits cerebrovascular dysfunction (via reduced vasodilator function as assessed by increasing doses of acetylcholine (Ach)), and cognitive dysfunction as AVP receptor antagonist SR49059 prevents these changes following 48 hours of water deprivation in rodents [20]. Hypohydration has also been demonstrated to increase plasma [endothelin-1] in both rodents [155] and humans [156]. This could be problematic as greater plasma [endothelin-1] has been associated with reduced peripheral vasodilatory function [157,158]. Interestingly, the neocortical application of endothelin receptor type A (ET_A_R) antagonist BQ123 ameliorates the cerebrovascular dysfunction induced by 48 hours of water deprivation in rodents [20]. Collectively, these data support a role for hypohydration influencing circulating factors that contribute to reduced blood vessel function.

### 3.6. Summary

There is a growing body of evidence that hypohydration induces inflammation, reduces endothelial function, and may affect measures of arterial stiffness in humans. Additionally, changes in several circulating factors during acute hypohydration may mediate changes in vascular function and BP regulation. The following sections will discuss the influence of acute hypohydration on cerebral blood flow regulation as well as BP regulation at rest, during orthostasis, and during exercise.

## 4. Cerebral Blood Flow Regulation

There are several reports of hypohydration being associated with worsened mood [18,89,90,91] and impaired cognitive function [19,20,21,91,92] that have prompted investigation into how acute hypohydration affects cerebral blood flow patterns. Tan et al. [87] had 10 male adults undergo magnetic resonance imaging (MRI) brain scans before running in ambient temperature (~25 °C) with a raincoat on to elicit a ~3% reduction in body mass on two occasions. Following exercise, participants either drank water to offset body mass loss or were restricted from fluid intake. During the second MRI brain scan 90 minutes after exercise, hypohydration produced reductions in total brain volume (total intracranial volume excluding ventricles) and increases in brain ventricular volume. However, there were no observed changes in global or regional brain perfusion, or functional activity of the brain during a motor-task based functional MRI (fMRI) scan during hypohydration [87]. Trangmar et al. [31] reported that incremental cycling exercise to exhaustion in the heat (35 °C) in 10 endurance-trained male adults elicited a ~3% reduction in body mass and lowered internal carotid and middle cerebral artery mean velocity (MCA_vmean)_ without affecting common carotid artery blood flow during exercise. However, when tested in the euhydrated state on a separate day, internal carotid and middle cerebral artery mean velocity and common carotid blood flow were preserved. This augmentation of hypohydration-induced decline in cerebral blood flow was reported to result from decreasing arterial carbon dioxide tension, which enhanced vasoconstrictor activity. Despite the reductions in cerebral blood flow, the cerebral metabolic rate for oxygen was maintained in the hypohydrated condition as a result of increased oxygen extraction [31]. Reductions in MCA_vmean_ and end-tidal carbon dioxide partial pressure (P_ET_CO_2_) have also been observed during a two-foot immersion cold pressor test in hypohydrated young male adults (~1% body mass loss via 24-hour fluid restriction) [159]. Together, these studies suggest that mild hypohydration in healthy young male adults is associated with alterations in cerebral blood flow regulation during acute sympathoexcitation (e.g., maximal exercise, the cold pressor test). Because hypohydration has been associated with reductions in cognitive function [19,20,21,92], more work in this area is warranted and future study designs should prioritize the inclusion of female adults.

## 5. Resting Cardiovascular Regulation

### 5.1. Sympathetic Nervous System 

Aside from promoting thirst and stimulating renal water reabsorption, signals of high central [Na^+^] are relayed to the rostral ventrolateral medulla (RVLM) and can affect BP through increases in sympathetic outflow [160,161,162]. During water deprivation in rats, blood hyperosmolality (i.e., elevated blood osmolality values) was found to influence sympathetic outflow and BP, independent of changes in plasma [AVP] and blood volume [163]. This is likely due to greater sensitivity of the PVN during times of blood hyperosmolality, demonstrated through studies using injections of γ-Aminobutyric acid (GABA) agonists and glutamate antagonists [32] and studies investigating changes to the intrinsic properties of RVLM neurons [160]. In support of these past reports, hypohydrated rats were found to have BP supported by PVN-driven increases in splanchnic sympathetic outflow that is not synchronized to changes in respiration or heart rate [161]. This is thought to occur from central hyperosmolality exciting discrete populations of neurons in the RVLM that increase sympathetic outflow and BP through the increased sensitivity of glutamate neurotransmission [162]. Importantly, alterations in sympathetic outflow and BP during central hyperosmolality are related to NaCl concentrations per se, as eqiu-osmotic sorbitol or mannitol does not produce the same OVLT neuronal discharge frequency [41]. Other animal studies suggest that activator protein-1 transcription factors are responsible for switching thoracic sympathetic outflow control from the hypothalamus to the commissural nucleus tractus solitarius (NTS) following water deprivation [164]. Blocking sympathetic outflow attenuates the BP elevations induced by high cerebrospinal fluid [Na^+^] in rodents [162]. Nonetheless, potential sensing mechanisms for [Na^+^] existing in the brain have been elucidated using rodent models [162,164,165,166]. A newly published study adds additional mechanistic insight, suggesting that Nax-positive glial cells in OVLT are activated by high [Na^+^], leading to enhanced hydrogen and lactate through a monocarboxylate transporter to activate ASIC1a-positive OVLT neurons [43]. More recently, one study in rodents demonstrated that sympathetic blockade (via α1- and β1- adrenergic receptor antagonists) significantly attenuated the increases in resting BP following 48 hours of water deprivation [165]. Together, these studies have provided insight into the role of the sympathetic nervous system activation to support BP during hypohydration.

### 5.2. Circulating Factors

Reduced sympathetic baroreflex function is associated with hypertension [166] and reduced cardiac vagal baroreflex sensitivity is associated with increasing age [167,168]. Importantly, reductions in baroreflex function can increase (i.e., worsen) BP variability, which is associated with cardiovascular morbidities such as cerebral small vessel disease [169], increased carotid artery intima-media thickness [170], target organ damage [117,171], hypertensive status [172], and cardiovascular mortality [119,120]. However, to date, only one study has investigated the influence of hypohydration on BP variability [173]. This study reported that iso-osmotic hypovolemia via furosemide (no body mass data reported) did not change the power spectral density of mean BP, a measure of BP variability in the frequency domain. One study in humans administered exogenous Ang II and observed increases in muscle sympathetic nerve activity (MSNA) [174]. Rabbitts et al. [28] used a 24-hour water restriction model in healthy young adults to elicit increases in endogenous [Ang II]. While body mass data following the water restriction protocol were not reported, resting MSNA burst incidence was reportedly increased with no change in resting BP. Despite increased MSNA burst incidence, both sympathetic and cardiac vagal baroreflex sensitivity were unchanged following water restriction [28]. This finding that water restriction in humans does not alter arterial baroreflex sensitivity is consistent with one previous study in 48-hour water-deprived rabbits [175]. Interestingly, in the human study, the water restriction-mediated increase in MSNA burst incidence was attenuated after the administration of losartan (an angiotensin receptor blocker), suggesting elevated [Ang II] produced endogenously provoked increases in sympathetic outflow [28]. Another study investigating the effects of hypohydration (~2% body mass loss) on baroreflex function noted a tendency for lower sympathetic baroreflex gain following hypohydration induced by 90 minutes of acute aerobic exercise compared to exercise and intravenous rehydration 20–25 minutes later [176]. While insightful, these data could have potentially been influenced by the prior bout of exercise (collected about 45–60 minutes post exercise). Together, these studies also report conflicting results regarding the influence of hypohydration on arterial baroreflex function. Thus, additional research would provide helpful insight. Further, research investigating the influence of hypohydration on BP variability is warranted.

During hypohydration, elevations in plasma [AVP] (tightly linked to changes in plasma osmolality [17]) and [Ang II] contribute to the maintenance of BP through numerous mechanisms [32,35,36,37,40,175,177]. For example, hypohydration in rats has been demonstrated to increase plasma renin activity, even during renal denervation and adrenal demedullation, suggesting sympathoadrenomedullary-independent plasma renin activity release to support BP [37]. When plasma renin activity is increased during hypohydration, angiotensin type-1 receptors in the PVN and RVLM are thought to become more sensitive [32], suggesting the renin–angiotensin–aldosterone axis mediates alterations in BP control through interaction with central cardiovascular control centers (i.e., the RVLM). Excessive AVP release has been suggested to play a role in glucoregulatory health [178] and in the development of human hypertension [179]. For a general review on the influence of AVP in cardiovascular control, the reader is directed to a review by Liard [180]. 

During hypovolemia, AVP is released via actions of the forebrain and midbrain [181] and supports BP by increasing lumbar sympathetic outflow and heart rate [163], independent of the involvement of the subfornical organ [182]. AVP blockade following water deprivation causes a significant drop in BP, suggesting its actions are necessary for BP support during water deprivation (WD) [177,183]. Rodent models using intravenous AVP antagonism demonstrate attenuated pressor and bradycardic effects of α1-adrenergic receptor agonists (e.g., methoxamine, phenylephrine) [184]. AVP antagonism in dogs attenuates the depressor and tachycardic effects of systemic nitric oxide-mediated vasodilation (via sodium nitroprusside), with no additive effect of Ang II antagonism, suggesting AVP plays a primary role in BP control during hypotensive insults [185]. In agreement, one study in rats demonstrated that the administration of intravenous synthetic AVP increases BP following water deprivation [40]. Further, Aisenbrey et al. [40] demonstrated that AVP blockade in rats lowers BP via reductions in peripheral vascular resistance, and this occurs independent of cardiac or arterial baroreceptor input [186]. In contrast, one previous study in rats reported that after 24 or 48 hours of water deprivation, AVP did not significantly contribute to BP maintenance [187]. There is also evidence that AVP only has a minor influence on BP support following hypohydration in humans (2% body mass loss via 24-hour fluid restriction), as selective V1 receptor antagonist [d(CH2)5Tyr(ME)]AVP elicited only minor reductions in diastolic BP and cardiac preload [188]. Together, the conflicting results in the literature regarding the influence of Ang II and AVP on BP regulation during hypohydration suggests more investigation in this area is necessary. Finally, several studies that have been conducted regarding the influence of biological sex [189,190,191,192,193,194,195,196,197,198] and sex hormone fluctuations during the menstrual cycle in female adults [189,191,192,193,194,195,196,197] and BP regulation. These studies have provided important insight concerning the influence of sex and menstrual cycle-induced changes in blood volume on BP regulation, which is a prerequisite to studying the additional influence of hypohydration. In summary, several circulating factors appear to influence resting BP regulation and the discrepancies in findings may be related to species differences as well as the method and degree of hypovolemia/hypohydration.

## 6. Cardiovascular Regulation During Orthostatic Stress

Orthostatic stress in humans occurs during daily life when posture changes from the supine or seated positions to the standing position. Approximately 500 mL of blood pools in lower body venous circulation immediately upon changing from the supine to the upright position [199]. To maintain BP and adequate cerebral perfusion upon standing, the body relies on rapid baroreflex-mediated increases in heart rate and MSNA. Without appropriate mechanisms to regulate BP during standing, there is an increased risk of syncope (i.e., fainting), which can result in an injury. While estimates vary among epidemiological studies, it has been reported that approximately ~10% of the population is orthostatic intolerant, defined as having significant drops in systolic and/or diastolic BP upon standing [200]. As a result of the obvious health concerns of syncope and head injuries, there has been a great amount of investigations aimed to determine the internal (i.e., physiological) and external (i.e., ambient temperature) factors that contribute to orthostatic intolerance because it is associated with adverse cardiovascular health outcomes [121,122]. For more details regarding the prognosis and treatment of orthostatic intolerance, the reader is directed to the following review article [201]. 

Experimentally, head-up tilt testing and lower body negative pressure (LBNP) challenges are commonly used to assess the integrated physiological responses that occur during orthostatic stress. The common factor among standing, head-up tilt testing, and LBNP is progressive central hypovolemia and, for this reason, head-up tilt testing [202] and LBNP [203,204] are valid models for assessing orthostatic tolerance, and can be affected by hydration status [30]. There are detailed reviews available that discuss the clinical applications of head-up tilt testing [205] and LBNP [206].

Related to hypohydration, one study from 1990 used furosemide (iso-osmotic hypovolemia) to elicit a ~2% body mass loss in healthy male adults. These participants demonstrated increased gain in cardiopulmonary baroreflex during a head-up tilt testing challenge (i.e., larger increase in vascular resistance for a given decrease in central venous pressure) [207]. Later, Cheuvront et al. [208] demonstrated that moderate hypertonic hypohydration (~5% body mass loss via exercise in the heat (40 °C)) and mild isotonic hypohydration (~3% body mass loss via furosemide), but not mild hypertonic hypohydration (~3% body mass loss via exercise in the heat (40 °C)), significantly increased sit-to-stand-induced changes in heart rate in healthy male and female adults. Work that is more recent has indicated that in response to a head-up tilt challenge, iso-osmotic hypovolemia (~3% body mass loss via furosemide) modulates heart rate and hyperosmotic hypovolemia (~3% body mass loss via exercise in the heat (40 °C)) modulates both heart rate and MSNA to support BP in healthy male and female adults [6]. Iso-osmotic hypovolemia via aldosterone receptor antagonist spironolactone (Aldactone; no body mass loss data reported) in healthy young males has been demonstrated to augment changes in total MSNA and total peripheral resistance during orthostasis to compensate for plasma volume (16% reduction) contraction-induced decrements in stroke volume and cardiac output [29]. A later published analysis of these data demonstrated that MSNA burst amplitude but not MSNA burst frequency mediated the observed increases in MSNA total activity during LBNP [209]. These studies demonstrate that plasma volume deficits imposed by hypohydration (e.g., reductions in plasma volume and increases in plasma osmolality) elicit alterations in the complex integrative cardiovascular responses that occur during an orthostatic challenge. Given that orthostatic intolerance is more common in female adults [200] and this research concerning hypohydration and cardiovascular responses to orthostatic challenges has been completed in young male adults, additional work in female adults and older populations are warranted. 

## 7. Cardiovascular Regulation During Exercise

Skeletal muscles require increased blood flow during exercise. Appropriate alterations in BP allow for increased blood flow to active skeletal muscle beds for the delivery of nutrients (e.g., oxygen) and for removal of metabolic byproducts (e.g., lactate). Augmented increases in BP during exercise is associated with greater future incidence of hypertension [124,125,126], as well as greater cardiovascular [123] and cardiometabolic [127] disease risk. Studies in rodents demonstrate that the hindbrain is responsible for mediating autonomic cardiovascular reflexes during hypovolemia to maintain BP [181]. Following 48 hours of water deprivation in rats, BP responses to unilateral RVLM microinjection of L-glutamate have been reported to be augmented, suggesting the increased sensitivity of RVLM neurons to excitatory amino acids during severe dehydration in rodents [210]. However, in our recent study, while 48 hours of WD in rodents increased resting lumbar sympathetic outflow and BP as previously reported in other studies [211], we did not observe water deprivation to change the responsiveness of sympathetic-regulatory neurons in the RVLM to the exogenous application of L-glutamate (sympathoexcitatory) or GABA (sympathoinhibitory) [212]. While the reasons for the discrepancies in findings between the former study [210] and our recent study [212] are unclear, we speculated that because injections were unilateral, intact compensatory contralateral pathways could have contributed to divergent observations. Nevertheless, additional work is warranted to provide insight into these autonomic cardiovascular responses following water deprivation.

In humans, moderate osmotic hypohydration (~5% body mass loss via cycling in the heat (35 °C)) has been demonstrated to attenuate the exercise-induced increases in BP, primarily by attenuating increases in cardiac output. One study in male adults demonstrated greater increases in heart rate and plasma [AVP] during exercise following mild hypohydration (3% body mass loss via cycling in the heat) versus 50 or 100% fluid replacement to offset body mass loss [134]. Additionally, these participants demonstrated accentuated increases in vascular resistance and plasma [norepinephrine], suggesting greater activation of the sympathetic nervous system during exercise in the hypohydrated state necessary to compensate for reductions in blood volume and pressure to maintain adequate skeletal muscle perfusion [5]. Recently, we sought to determine whether mild hypohydration affects sympathetic and BP responses during exercise pressor reflex activation. We found that very mild hypohydration (~0.5% body mass loss) did not affect MSNA or BP responses during static handgrip exercise in healthy young male and female adults [212]. While the observed changes in body mass were modest following voluntary reductions in water intake over three days concluded with a 16-hour water abstention period, key considerations in our study design were to elicit increases in serum [Na^+^] and determine the resultant alterations in exercise pressor reflex function. Additionally, this study design allowed for a hypohydration stimulus in the absence of exercise, heat, and diuretic usage. It is possible that a combination of methods is required to produce more severe hypohydration and elicit alterations in exercise pressor reflex function.

## 8. Cardiovascular Regulation and Body Water Balance During Hypobaric Hypoxia

Acute hypobaric hypoxia (i.e., high-altitude) increases BP [213] and alters body water balance via fluid shifts and changes in hormonal control of body fluid and electrolytes [214,215,216]. The increases in BP during acute exposure to altitude is thought to occur through endothlin-1-mediated increases in heart rate and systemic sympathetic activation. With chronic altitude exposure, there is potential to develop chronic arterial and pulmonary hypertension, the mechanisms and evidence for which are discussed in depth by Riley et al. [213]. Specific to changes in body water balance, acute altitude exposure (3500 m for 12 days) elicits reductions in extracellular water and total body water [214]. In agreement with this observation, another study reported that during the first three days at an elevation of 5334 m, plasma volume and total body water were reduced, while plasma renin activity and serum [aldosterone] increased. As expected with these observations, sodium and potassium excretion were concomitantly reduced [216]. The findings from these previous studies are consistent with other work that demonstrated dehydration upon arrival to 4850 m was induced by fluid shifts to the interstitial space and produced rapid hemoconcentration (i.e., increases in hemoglobin concentrations and hematocrit values). The authors speculated that any further hemoconcentration observed during the climb from 4850 m to 7600 m can be partially explained by stimulated erythropoiesis [215]. To summarize, acute hypobaric hypoxia elicits alterations in body water balance that produce unfavorable conditions for optimal physiological function. While extended discussion on strategies to mitigate the deleterious effects of altitude on physiological function is beyond the scope of this review article, the authors suggest a review [85] by Sawka and colleagues for more information on this topic.

## 9. Summary

Hypohydration is known to reduce mental and physical performance, and more recent evidence suggests hypohydration also impairs vascular function and cardiovascular regulation. Specifically, hypohydration has been demonstrated to impair cutaneous vascular function, reduce endothelial function, and alter BP regulation at rest during exercise and during orthostatic stress (Figure 1). Future studies examining the physiological effects of hypohydration in healthy female adults are warranted as most of the previous work has been completed within male adults. Additionally, studies determining the acute and chronic effects of hypohydration in preclinical populations, such as old adults and those with hypertension, are warranted.

## 10. Perspectives

Previous literature indicates that mild hypohydration impairs cognitive function, aerobic exercise performance, and thermoregulation. Here, we highlighted the negative implications of hypohydration on vascular function and cardiovascular regulation at rest and during various perturbations (e.g., orthostatic stress, exercise). While there is less consensus regarding more mild forms of hypohydration on these cardiovascular measures, there is strong evidence that mild-to-moderate hypohydration impairs several indices of cardiovascular function. Taken together, these studies indicate that acute reductions in water intake may negatively influence cardiovascular function in healthy young humans. These deleterious cardiovascular effects of mild hypohydration are more consistent during protocols that employ exercise, heat stress, and/or diuretic usage in addition to water restriction.
